# Nomogram for Predicting the Risk of Short Sleep Duration in Myocardial Infarction Survivors

**DOI:** 10.31083/j.rcm2503077

**Published:** 2024-02-28

**Authors:** Jun Xu, Gang Qin

**Affiliations:** ^1^First School of Clinical Medicine, Shanxi Medical University, 030000 Taiyuan, Shanxi, China; ^2^Department of Cardiology, First Hospital of Shanxi Medical University, 030000 Taiyuan, Shanxi, China

**Keywords:** myocardial infarction, short sleep duration, nomogram, risk factors, NHANES database

## Abstract

**Background:**

Research on post-infarction insomnia, particularly short sleep duration following myocardial infarction (MI), remains limited. Currently, there are no existing guidelines or risk prediction models to assist physicians in managing or preventing short sleep duration or insomnia following MI. This study aims to develop a nomogram for predicting the risk of short sleep duration after MI.

**Methods:**

We conducted a retrospective study on 1434 MI survivors aged 20 and above, utilizing data from the National Health and Nutrition Examination Survey (NHANES) database spanning from 2007 to 2018. Among them, 710 patients were assigned to the training group, while 707 patients were allocated to the testing group. We utilized logistic regression, least absolute shrinkage and selection operator (LASSO) regression, and the elastic network for variable selection. The stability and accuracy of the prediction model were assessed using receiver operator characteristics (ROCs) and calibration curves.

**Results:**

We included five variables in the nomogram: age, poverty income ratio (PIR), body mass index (BMI), race, and depression. The ROC curves yielded values of 0.636 for the training group and 0.657 for the testing group, demonstrating the model’s good prediction accuracy and robustness through a calibration curve test.

**Conclusions:**

Our nomogram can effectively predict the likelihood of short sleep duration in MI survivors, providing valuable support for clinicians in preventing and managing post-MI short sleep duration.

## 1. Introduction

Myocardial infarction (MI) is a series of events that includes myocardial ischemia and necrosis events, resulting from insufficient myocardial blood supply due to vascular blockage [1]. Subsequently, survivors of an MI often experience multiple post-infarction symptoms. Approximately 55% of patients display varying degrees of sleep disorders four months after MI, which can impede cardiac recovery [2]. Notably, clinical observations have revealed that insomnia, including difficulty staying asleep, is prevalent in individuals post-MI [3]. Furthermore, MI patients with short sleep durations have a higher incidence of major adverse cardiovascular events (MACEs) compared to those without sleep disorders [4, 5].

Sleep plays an important role in maintaining overall health and daily functioning [5]. Approximately 22% of the global population experiences insomnia symptoms [6], which typically include difficulty falling asleep, trouble sustaining sleep, which means waking up frequently or prematurely during the night, and waking up too early in the morning [7, 8, 9]. The prevalence of insomnia is increasing [10]. Insomnia can lead to the development of MI, and MI can lead to the development of insomnia [11, 12, 13]. Prolonged and reduced sleep durations are both linked to higher mortality rates, and the presence of insomnia symptoms further exacerbates deaths related to cardiovascular disease [14, 15]. These conditions significantly reduce the quality of life and impose a substantial disease burden.

Current research indicates that insomnia is associated with endocrine, metabolic, cortical function, and neurological disorders [16], as well as risk factors such as lack of exercise and an irregular diet [17]. Insomnia is also correlated with body mass index (BMI), hypertension, anxiety and depression [18, 19, 20].

Recognizing patients predisposed to post-infarction insomnia early and providing timely interventions is crucial. However, the current literature on post-infarction insomnia, especially concerning short sleep duration after MI, is limited. Consequently, there is a lack of existing guidelines or risk prediction models to assist physicians. Developing a numerically-scored risk identification system for post-MI short sleep duration would significantly improve patients’ life quality. This study, utilizing the National Health and Nutrition Examination Survey (NHANES) database, aims to establish a risk prediction method for post-MI short sleep duration by examining relevant factors.

## 2. Methods

### 2.1 Data Source

The data for this retrospective analysis were obtained from the NHANES database, including six survey cycles ranging from 2007 to 2018. NHANES is a survey sponsored by the Centers for Disease Control (CDC) and National Center for Health Statistics (NCHS). Survey participants provided informed consent and underwent ethical review approval from NCHS before receiving the questionnaire and examination. This study was conveniently exempt from further ethical scrutiny [21].

### 2.2 Study Population

A total of 1434 survivors of acute MI were obtained by selecting data from adults aged 20 and above for the study. However, 9 individuals lacking the sleep questionnaire data and 8 individuals with incomplete data on depression were excluded. As a result, the study included 1417 survivors of acute MI. We allocated 710 patients from 2007 to 2012 to the training group and 707 patients from 2013 to 2018 to the testing group.

### 2.3 Data Collection

The response variable in this study was short sleep duration, defined as self-reported sleep of less than six hours daily [22]. We collected thirteen potential predictor variables (Table 1). Demographic data, such as age, gender, race, education, and marital status, were obtained from self-administered questionnaires. BMI was calculated from weight divided by height squared (kg/$m^{2}$) during the interview. Diabetes status was determined based on glycosylated hemoglobin levels from the laboratory data, with a value ≥6.5% considered as diabetes. Smoking, drinking, exercise, depression screening, and hypertension were self-reported by the participants. Subjects who reported drinking more than 10 days in the past year and consuming more than 5 drinks per day were considered severe drinkers, those who drank less than 5 drinks per day for less than 10 days in the past year were considered moderate drinkers, those who drank less than 12 drinks in their lifetime were considered as non-alcoholic drinkers, and others were considered mild drinkers [23]. In a typical week, their exercise status was determined based on the number of days the subject engaged in vigorous intensity sports, fitness, or regenerative activities. The poverty income ratio (PIR) was determined according to the standards set by the Department of Health and Human Services (HHS), which vary based on household size and state.

**Table 1.** **Participant characteristics**.

Characteristics		All	Train	Test	*p* value
Number		1417	710	707	
Age (years)		66.56 ± 12.10	66.26 ± 12.57	66.85 ± 11.63	0.042
PIR		2.05 ± 1.47	2.05 ± 1.48	2.06 ± 1.46	0.968
BMI (kg/$m^{2}$)		30.27 ±7.05	30.28 ± 6.63	30.27 ± 7.45	0.147
Gender (n, %)					0.574
	Man	940 (66.30)	476 (67.00)	464 (65.60)	
	Woman	477 (33.70)	234 (33.00)	243 (34.40)	
Race (n, %)					0.074
	Mexican American	141 (10.00)	68 (9.60)	73 (10.30)	
	Other Hispanic	126 (8.90)	60 (8.50)	66 (9.30)	
	Non-Hispanic White	779 (55.00)	419 (59.00)	360 (50.90)	
	Non-Hispanic Black	261 (18.40)	127 (17.90)	134 (19.00)	
	Other Race-Including Multi-Racial	110 (7.80)	36 (5.10)	74 (10.50)	
Education (n, %)					<0.001
	Below high school	489 (34.50)	286 (40.30)	203 (28.70)	
	High school	362 (25.50)	173 (24.40)	189 (26.70)	
	Above high school	566 (39.90)	251 (35.40)	315 (44.60)	
Marital status (n, %)					0.244
	Living with a partner	786 (55.50)	405 (57.00)	381 (53.90)	
	Single	538 (38.00)	260 (36.60)	278 (39.30)	
	Never married	93 (6.60)	45 (6.30)	48 (6.80)	
Hypertension (n, %)					0.533
	No	361 (25.50)	186 (26.20)	175 (24.80)	
	Yes	1056 (74.50)	524 (73.80)	532 (75.20)	
Diabetes (n, %)					0.602
	No	1013 (71.50)	512 (72.10)	501 (70.90)	
	Yes	404 (28.50)	198 (27.90)	206 (29.10)	
Drinking (n, %)					<0.001
	No	543 (38.30)	320 (45.10)	223 (31.50)	
	Light	734 (51.80)	309 (43.50)	425 (60.10)	
	Moderate	127 (9.00)	74 (10.40)	53 (7.50)	
	Heavy	13 (0.90)	7 (1.00)	6 (0.80)	
Smoking (n, %)					0.369
	No	481 (33.90)	233 (32.80)	248 (35.10)	
	Yes	936 (66.10)	477 (67.20)	459 (64.90)	
Exercise (n, %)					0.222
	No	1005 (70.90)	514 (72.40)	491 (69.40)	
	Yes	412 (29.10)	196 (27.60)	216 (30.60)	
Depression (n, %)					0.973
	No	1174 (82.90)	588 (82.80)	586 (82.90)	
	Yes	243 (17.10)	122 (17.20)	121 (17.10)	
Short sleep duration (n, %)					<0.001
	No	903 (63.70)	397 (55.90)	506 (71.60)	
	Yes	514 (36.30)	313 (44.10)	201 (28.40)	

PIR, poverty income ratio; BMI, body mass index.

### 2.4 Statistical Analysis

The data in this study were weighted according to NHANES guidelines. Continuous variables are represented as mean ± standard deviation, while the categorical variables are expressed as count (%). Group comparisons were conducted using the Student’s *t*-test for continuous variables and the Wilcoxon test for categorical variables. In SPSS software (Version 25.0, IBM Corp., Armonk, NY, USA), univariate and multivariate logistic regression analyses were performed to screen variables and diagnose collinearity. Odds ratio (OR) and 95% confidence interval (CI) were used as effect estimates. Using the least absolute shrinkage selection operator (LASSO) regression and cross-validation in R software (version 4.4.2, R Foundation for Statistical Computing, Vienna, Austria) to determine optimal predictor variables. The nomogram’s predictive efficacy was evaluated using the receiver operator characteristic (ROC) curve, and calibration curves were generated to assess the accuracy of the predictive model. *p* < 0.05 was considered statistically significant.

## 3. Result

### 3.1 Baseline Clinical Characteristics

As shown in Table 1, a total 1417 myocardial infarction survivors were involved in the study. Of these, 940 were men (66.30%), and 477 were women (33.70%). The participants were divided into two groups, with 710 in the training group and 707 in the testing group. The response variable was short sleep duration, and the remaining 13 variables were considered as candidate predictors. Among these participants, a total of 514 (36.30%) suffered from short sleep duration, while 903 (63.70%) did not.

### 3.2 Association between Predictive Variables and Short Sleep Duration

Table 2 presents the results of logistic regression analysis for predictive variables. Univariate logistic regression revealed a significant positive relationship between depression and short sleep duration (OR: 1.855, 95% CI: 1.403–2.452). Subsequently, seven significant variables from the univariate analysis were chosen for multivariate logistic regression. The results indicated varying risks of short sleep duration among different races. Notably, the Non-Hispanic Black individuals showed the highest disparity (OR: 2.545, 95% CI: 1.612–4.016). As age increased, the risk of short sleep duration decreased (OR: 0.979, 95% CI: 0.967–0.988). Simultaneously, a higher risk was observed among patients with depression (OR: 1.479, 95% CI: 1.098–1.993).

**Table 2.** **Univariate and multivariate logistic regression screen candidate variables**.

Variable		Univariate		Multivariate	
		OR (95% CI)	*p* value	OR (95% CI)	*p* value
Age		0.973 (0.964, 0.982)	<0.001	0.979 (0.967, 0.988)	<0.001
PIR		0.815 (0.753, 0.881)	<0.001	0.869 (0.797, 0.948)	0.002
BMI		1.020 (1.005, 1.036)	0.011	1.010 (0.994, 1.026)	0.219
Race					
	Mexican American	1.0		1.0	
	Other Hispanic	1.721 (1.030, 2.875)	0.038	1.778 (1.052, 3.005)	0.032
	Non-Hispanic White	1.325 (0.890, 1.973)	0.165	1.644 (1.086, 2.489)	0.019
	Non-Hispanic Black	2.404 (1.545, 3.739)	<0.001	2.545 (1.612, 4.016)	<0.001
	Other Race-Including Multi-Racial	1.380 (0.805, 2.366)	0.241	1.535 (0.880, 2.677)	0.131
Education					
	Below high school	1.0		1.0	
	High school	0.964 (0.730, 1.274)	0.798	0.974 (0.724, 1.309)	0.860
	Above high school	0.739 (0.574, 0.952)	0.019	0.855 (0.643, 1.138)	0.283
Exercise					
	No	1.0		1.0	
	Yes	0.769 (0.603, 0.980)	0.034	0.897 (0.691, 1.163)	0.411
Depression					
	No	1.0		1.0	
	Yes	1.855 (1.403, 2.452)	<0.001	1.479 (1.098, 1.993)	0.010
Gender					
	Male	1.0			
	Female	1.209 (0.963, 1.518)	0.102		
Marital status					
	Living with a partner	1.0			
	Single	1.135 (0.904, 1.425)	0.276		
	Never married	1.121 (0.719, 1.749)	0.614		
Hypertension					
	No	1.0			
	Yes	1.083 (0.844, 1.391)	0.530		
Diabetes					
	No	1.0			
	Yes	0.992 (0.780, 1.261)	0.947		
Drinking					
	No	1.0			
	Light	0.827 (0.656, 1.043)	0.109		
	Moderate	1.442 (0.997, 2.129)	0.066		
	Heavy	1.939 (0.643, 5.849)	0.240		
Smoking					
	No	1.0			
	Yes	1.108 (0.880, 1.394)	0.383		

PIR, poverty income ratio; BMI, body mass index; OR, odds ratio.

### 3.3 Predicting Short Sleep Duration in the Training and Testing Groups

**Fig. 1.**
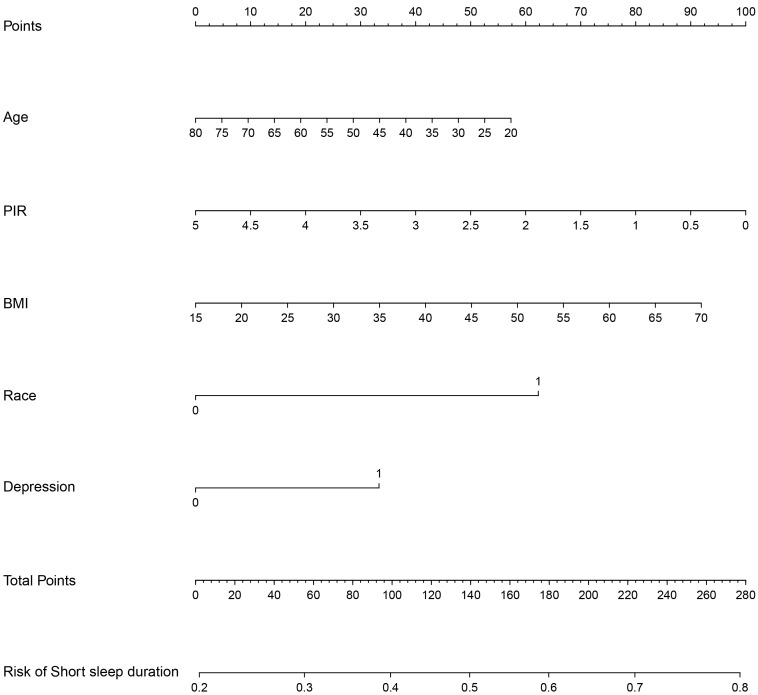
**A nomogram for predicting short sleep duration risk in the training group**. PIR, poverty income ratio; BMI, body mass index.

We developed a prediction model that incorporates five distinct variables based on their clinical relevance, scientific insights, multivariate regression outcomes, and findings from prior studies. The nomogram, depicted in Fig. 1, includes age, PIR, BMI, race, and depression. A collinearity check amongst these five predictors confirmed no collinearity issues. To simplify interpretation and due to the pronounced significance of the Non-Hispanic Black category, we reclassified races into two groups: Non-Hispanic Black and Other races. The nomogram assigns scores based on variable values, enabling straightforward assessment of the likelihood of short sleep duration post-myocardial infarction for individual patients.

**Fig. 2.**
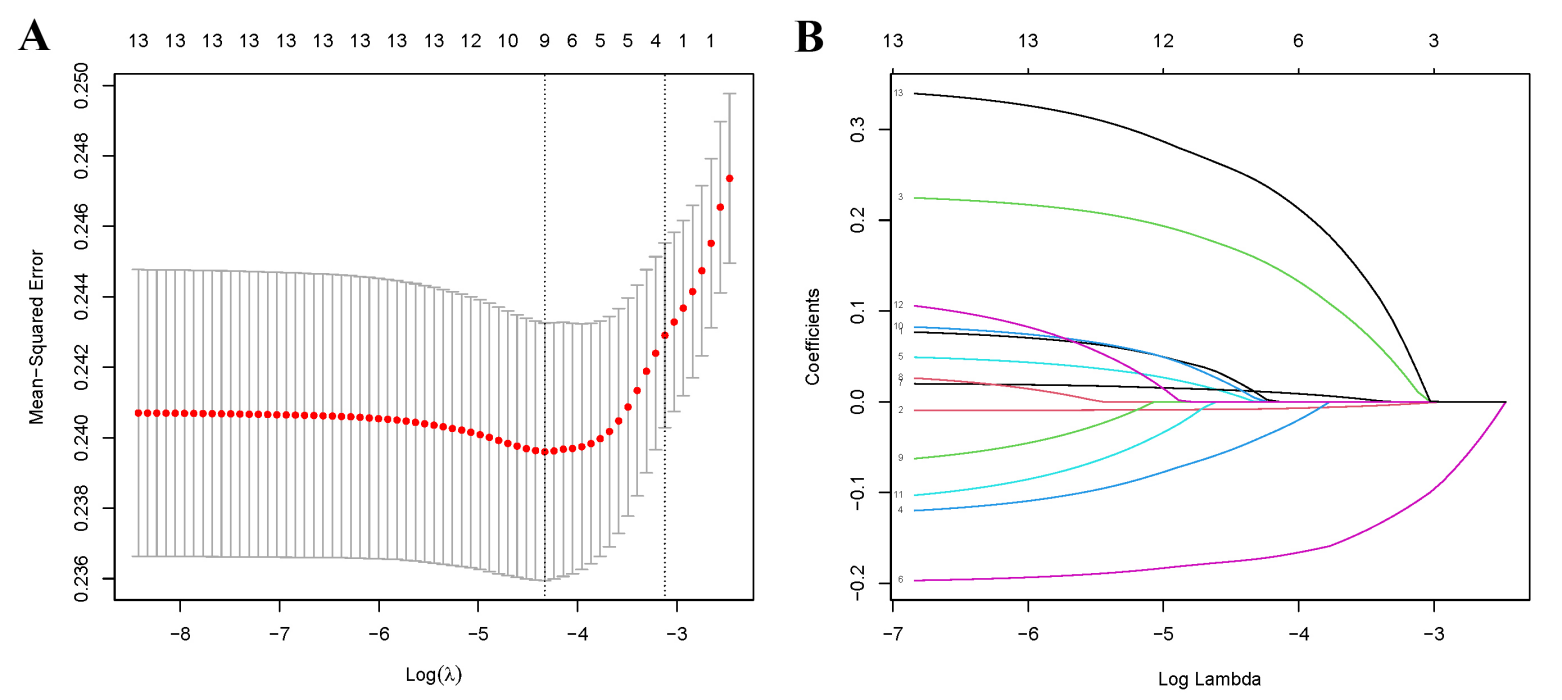
**The LASSO binary regression and cross validation method for screening variables**. (A) The relationship between Mean-Square Error and Log (lambda). (B) LASSO-derived coefficient distribution for the 13 variables. LASSO, least absolute shrinkage and selection operator.

Furthermore, we employed the LASSO binary regression combined with cross-validation to refine our variable selection. Fig. 2A illustrates the relationship between Mean-Squared Error and log (lambda), with dashed lines marking the minimum criterion and the minimum criterion plus a standard error. Fig. 2B illustrates the coefficients of the 13 variables against log (lambda). An optimal lambda value was determined using a standard error value of 0.04418081. The chosen variables—age, race, PIR, and depression—aligned with results from the multivariate logistic regression. When the LASSO binary regression was applied to five variables, BMI also emerged. Mean squared errors for both four and five variables were 0.2243591 and 0.2222506, respectively. Given the marginal difference in error, we chose to include all five variables.

**Fig. 3.**
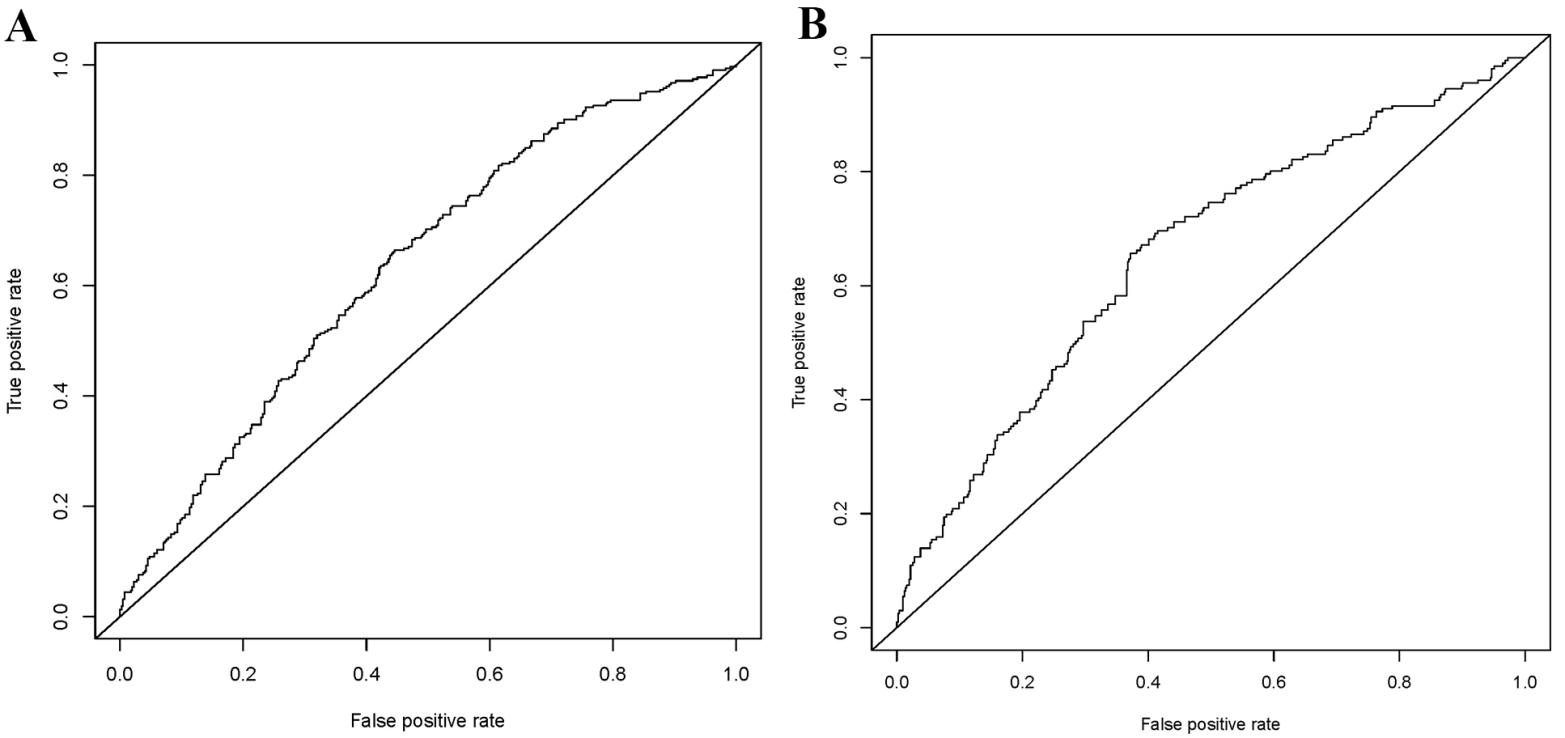
**Receiver operating characteristic (ROC) curves for the training (A) and testing (B)**.

**Fig. 4.**
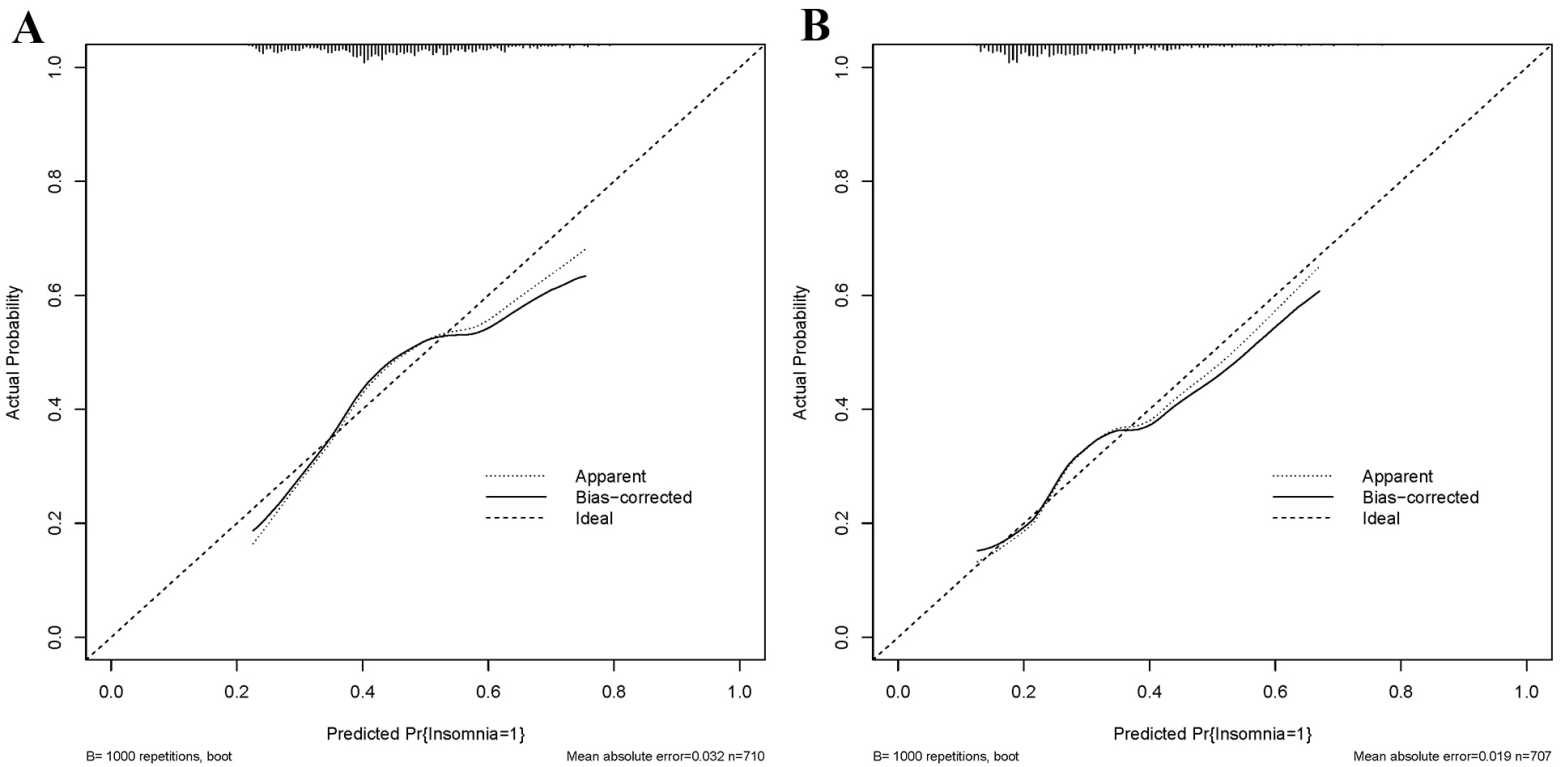
**Calibration curves for the training (A) and testing (B) groups**.

As shown in Fig. 3, the C-statistic for the training group was 0.636 (Fig. 3A), while that for the testing group was 0.657 (Fig. 3B), indicating the model’s consistency. The calibration curves are displayed in Fig. 4. The diagonal line represents the ideal prediction result, while the solid line represents the actual performance of the prediction model [24]. It is worth noting that the model exhibited good predictive performance in both groups.

## 4. Discussion

To date, a risk prediction method targeting short sleep duration following MI has not been previously developed. This study pioneers this field by utilizing data from 1417 MI survivors sourced from the NHANES database.

We found that patients with post-MI depression were more likely to have a short sleep duration. Similarly, age exhibited an inverse correlation with short sleep duration. Moreover, our analysis revealed a notable observation: MI survivors who engage in regular weekly exercise tend to have a decreased risk of short sleep duration. Interestingly, while many studies have emphasized the role of health conditions like hypertension and diabetes in determining sleep patterns, our dataset did not identify any significant variance in sleep duration based on these conditions. Another intriguing observation is the negative association of PIR with short sleep duration, post-MI. This suggests a potential socioeconomic dimension to the issue, indicating that individuals with lower income brackets might be at a greater risk [24]. Interestingly, there were statistically significant differences in short sleep duration after myocardial infarction among races. Specifically, compared to Mexican Americans, Non-Hispanic Black individuals showed the highest risk. While we categorized race into two broader categories for ease of interpretation in the nomogram, future research might benefit from a more nuanced approach, assigning unique scores to each racial group to enhance prediction accuracy. Despite gender being a significant factor in many health outcomes, our data did not delineate a notable difference between genders in terms of post-MI sleep duration. This finding is contrary to earlier research, which identified gender-specific causes for MI [25].

To enhance the precision of our variable selection, we incorporated the LASSO binary regression and cross-validation techniques. The alignment between this approach and binary logistic regression fortified the credibility of our predictive model. The area under the curve (AUC) in the training group was 0.636, and in the testing group was 0.657, indicating the good stability of the model. The calibration curve further affirmed the model’s accuracy in predicting short sleep duration after myocardial infarction. Given that most of the predictor variables included in the model are objective demographic characteristics, along with the PIR having geographic distribution characteristics, we assumed that the predicted outcome will not be influenced by the subjective feelings of the physician or patients. The Patient Health Questionnare-9 (PHQ-9), a primary depression questionnaire, is widely recognized for its validity [26]. Due to database limitations, we did not include patient biochemical indicators, inflammatory markers, and other data. However, our predictive model still demonstrated good predictive performance, which can help doctors in identifying whether patients are likely to experience short sleep duration after myocardial infarction in advance, ultimately improving their quality of life. Furthermore, it is important to note that the pathogenesis of short sleep duration after myocardial infarction is limited to the reasons mentioned above and remains a subject for further study. The sensitivity of patients’ responses to stress after MI varies, as does the timing of the onset of short sleep duration [13]. Therefore, the assessment of short sleep duration risk in post-MI patients should be dynamic. A nomogram score of 0 does not mean that a patient will not experience short sleep duration, and this possibility should not be ignored.

Studies have shown that patients who sleep less than six hours are at increased risk of developing hypertension, experiencing acute myocardial infarction, heart failure, and higher morbidity and mortality rates [27]. In fact, sleeping for less than six hours at night has been linked to an increased risk of cardiovascular diseases in multiple studies [28, 29, 30]. In this study, sleep of less than 6 hours was specifically defined as short sleep duration [22, 31]. Interestingly, in European countries, the initial goal of treatment for patients with short sleep duration is to achieve 6.5 hours [32]. However, it has also been noted that short sleep duration, such as ≤5 hours, has also been strongly associated with myocardial infarction [33].

However, it is imperative to acknowledge the limitations of our study. The symptoms of short sleep duration include difficulty falling asleep, frequent waking up during the night, and premature awakening. Early awakening indicates a lack of sleep time. In this study, we focused on the situation of insufficient sleep time due to the limitations of the sample data, preventing us from obtaining more detailed information about the sleep status of the subjects. In the subsequent clinical study, assessment tools like the Jenkins Sleep Scale (JSS)-4 [34], the Pittsburgh Sleep Quality Index (PSQI) [35] and the Bergen Short sleep duration Scale (BIS) [36] can be used to comprehensively evaluate the sleep status of individual patients. Furthermore, it is important to acknowledge that there are numerous causes of short sleep duration, and insufficient sleep time may be a result of either short sleep or reduced sleep duration. Unfortunately, we were unable to obtain detailed reasons for each participant’s insufficient sleep time from the questionnaire. Based on these limitations, future research should aim for greater refinement to investigate the underlying causes of short sleep duration after myocardial infarction and to develop strategies for its prevention and intervention.

Highlighting the importance of disease prevention is equally, if not more critical than focusing solely on treatment. Patients suffering from diseases such as hypertension, coronary heart disease, and myocardial infarction not only endure physical pain but also experience a serious psychological burden as a result of their illness. It is expected to find appropriate drugs for preventing or treating short sleep duration after an MI to achieve a dual benefit. This is evident in the connection between hypoglycemic drugs and cardiovascular diseases. The therapeutic role of anti-diabetic medications like metformin is manifested through the reduction of cardiac pump deterioration and heart failure events [37]. The effect is achieved via their systemic and local anti-inflammatory properties [38], as well as in patients with non-obstructive coronary stenosis and endothelial dysfunction [39]. Notably, sodium–glucose transporter 2 inhibitors (SGLT2i) have recently demonstrated beneficial effects on endothelial dysfunction [40]. They play a role in stabilizing atherosclerotic coronary plaques and reducing adverse cardiac events following myocardial infarction [41, 42]. Additionally, for post-myocardial infarction patients experiencing short sleep duration, detecting and intervening in patients with a mental health condition in a timely manner during the diagnosis and treatment process are crucial. Whenever possible, a single drug treatment can help reduce the medication burden on patients, ultimately improving clinical outcomes in patients with acute myocardial infarction, thus ensuring the best possible treatment for the patients.

## 5. Conclusions

We innovatively proposed a model to predict the risk of short sleep duration after MI, based on the demographic information of patients and the characteristics of self-reporting. Our nomogram can predict the probability of myocardial infarction survivors with short sleep duration, helping clinical doctors prevent and intervene in the occurrence of short sleep duration after MI.

## Data Availability

The datasets used and analyzed during the current study are available from the corresponding author on reasonable request.
